# Mitochondrial Trifunctional Protein Deficiency due to *HADHA* Variants Masquerading as Charcot–Marie–Tooth Disease

**DOI:** 10.1111/jns.70048

**Published:** 2025-08-11

**Authors:** Farkhanda Qaiser, John McHugh, Gerard Mullins, Michael Farrell, Loai Shakerdi, James O. Byrne, Sinéad M. Murphy

**Affiliations:** ^1^ Department of Neurology Tallaght University Hospital Dublin Ireland; ^2^ Department of Neurophysiology Tallaght University Hospital Dublin Ireland; ^3^ Department of Neurophysiology Beaumont Hospital Dublin Ireland; ^4^ Department of Neuropathology Beaumont Hospital Dublin Ireland; ^5^ National Centre for Inherited Metabolic Disorders Mater Misericordiae University Hospital, Eccles St. Dublin Ireland; ^6^ School of Public Health University College Dublin Dublin Ireland; ^7^ Academic Unit of Neurology Trinity College Dublin Dublin Ireland

**Keywords:** Charcot–Marie–Tooth disease (CMT), HADHA, inherited neuropathy, mitochondrial trifunctional protein deficiency (MTPD), neuromyopathic

## Abstract

**Background and Aims:**

Mitochondrial trifunctional protein deficiency (MTPD) is an inherited disorder of fatty acid β‐oxidation caused by mutations in *HADHA* or *HADHB* genes. It typically presents with cardiomyopathy or hepatic failure in early childhood; however, it may rarely present in adulthood with the neuromyopathic form.

**Methods:**

We describe a patient with MTPD with isolated neuropathy mimicking Charcot–Marie–Tooth disease (CMT) as the first and only presenting symptom. Clinical and electrophysiological examinations were conducted, including nerve conduction studies, needle electromyography, muscle and nerve biopsies. The diagnosis was confirmed with genetic testing and enzymatic analysis of cultured skin fibroblasts.

**Results:**

We report a 40‐year‐old man diagnosed with axonal CMT2 in childhood. He had pes cavus and hammer toes, mild distal lower limb weakness, and loss of vibration sense with areflexia. He later developed fatigability, improved exercise tolerance with alcohol and an episode of chest infection causing neurological decompensation without evidence of rhabdomyolysis. Neurophysiology showed non‐length‐dependent axonal sensorimotor neuropathy without myopathic features. Genetic testing confirmed that he was compound heterozygous for two *HADHA* variants, one of them novel, and enzymatic analysis of cultured skin fibroblasts confirmed MTPD.

**Interpretation:**

We report a very rare isolated neuropathic phenotype of MTPD and confirm the pathogenicity of the novel variant c.1003G>A, p.(Glu335Lys). This case also highlights the need for *HADHA* and *HADHB* to be included in neuropathy gene panels as MTPD may present as CMT. Given that dietary management may prevent some complications of MTPD, achieving a diagnosis early is important.

## 1 Background and Aims

Long chain 3‐hydroxyacyl CoA dehydrogenase deficiency (LCHADD) and mitochondrial trifunctional protein deficiency (MTPD) are rare autosomal recessive disorders of fatty acid β‐oxidation. MTP is an enzyme complex consisting of four α and four β subunits encoded by *HADHA* and *HADHB*, respectively [1]. The α subunits contain the enzymes long chain enoyl CoA hydratase (LCEH), LCHAD and the β subunits contain long chain ketoacyl CoA thiolase (LCKT).

LCHADD due to isolated deficiency of LCHAD is more common, caused by *HADHA* variants, whereas MTPD due to deficiency of all three enzymes is much rarer and caused by *HADHA* or *HADHB* variants. Both of these conditions are part of newborn screening (NBS) in about 20 European countries but not in Ireland.

MTPD was first described in 1992 and may manifest with diverse clinical phenotypes including a severe neonatal form with cardiomyopathy, an infantile hepatic type and a milder late‐onset neuromyopathic phenotype. The neuromyopathic presentation is the least common, presenting with repeated episodes of rhabdomyolysis, usually after metabolic decompensation, along with progressive peripheral sensory‐motor neuropathy. Spiekerkoetter et al. described 11 patients with a neuromyopathic phenotype and reported that the combination of episodic rhabdomyolysis and peripheral neuropathy was present in almost all patients [2]. In another small study, 70% of patients had neuropathy as a long‐term complication [3].

We present a patient with MTPD due to compound heterozygous *HADHA* variants, one of them novel, with childhood onset isolated neuropathy initially diagnosed as Charcot–Marie–Tooth disease (CMT). While superimposed exacerbations occurred, there was no documented evidence of rhabdomyolysis or metabolic decompensation.

## 2 Case Report

This 40‐year‐old male walked late at 2 years and toe‐walked in early childhood. He had poor balance and was always slow at running. He was first seen by neurology services at 6 years when he was documented to have high arches, toe walking and diminished reflexes without sensory loss. Neurophysiology confirmed an axonal neuropathy, and he was given a diagnosis of Charcot–Marie–Tooth disease type 2 (CMT2). At 8 years, Achilles tendon lengthening was performed. In his early teens, he developed some distal upper limb symptoms with difficulty playing the guitar and typing. He did not report permanent functionally limiting weakness but described episodic limb weakness and fatigability with prolonged activity, which resolved with resting, occasionally associated with distal sensory symptoms. He consistently reported improved exercise tolerance after drinking alcohol; thus, he regularly drank alcohol in order to improve his functional ability before doing any activity. At 30 years, following a respiratory tract infection, he developed gradual and progressive weakness, eventually being unable to lift his head off his chest, requiring hospital admission. Examination during this admission documented pes cavus and hammer toes, neck flexion weakness, proximal more than distal weakness in all four limbs (weakest being shoulder abduction and hip flexion, MRC grade 3) with areflexia and distal loss of vibration sense. With only supportive management, symptoms gradually improved over the following 3 months, but he was left with mild residual distal weakness.

Examination at 40 years revealed mild distal lower limb weakness (ankle dorsiflexion MRC grade 4) and loss of vibration sense with areflexia. There was no pigmentary retinopathy.

There was no family history of neuromuscular or metabolic disorder, and no consanguinity.

Investigations performed during his hospital admission at 30 years included CSF, which was acellular, protein 51 mg/dL (15–45) and glucose 3.4 mmol/L. CK 625 U/L (55–170). Septic screen was performed and revealed no signs of infection. Routine blood tests were unremarkable. Other investigations were normal or negative, including autoantibodies, serum protein electrophoresis, very long chain fatty acids, vitamin E, lactate, pyruvate, ammonia, urinary porphyrins, urinary glycosaminoglycans, organic acids, amino acids, acylcarnitine profile and free carnitine. MRI whole spine showed no significant abnormality.

Nerve conduction studies at 30 years (Table 1) showed reduced compound muscle action potential (CMAP) and sensory nerve action potential (SNAP) amplitudes in a non‐length‐dependent pattern with normal conduction velocities. Needle electromyography showed an excess of polyphasic units of long duration in all muscles sampled, with reduced recruitment and rapid firing suggesting re‐innervation. There were also chronic neuropathic features in all muscles tested, including bulbar muscles and genioglossus. There were no myopathic features and no decrement on repetitive nerve stimulation. Overall, the findings were in keeping with a non‐length‐dependent motor‐predominant axonal neuropathy. These were repeated at 35 years, and the findings were similar.

**TABLE 1:** Nerve conduction studies, abnormal values in bold.

Motor nerves	Latency (ms)	Amplitude (mV)	Conduction velocity (m/s)
Right Median			
Wrist—APB	3.8	**4.2**	
Elbow—wrist	9.0	**3.1**	50.0
Left Median			
Wrist—APB	**4.2**	**3.6**	
Elbow—wrist	9.2	**2.9**	54.0
Right Ulnar			
Wrist—ADM	3.3	**4.4**	
Bel elb—wrist	7.8	**4.4**	58.0
Ab elb—bel elb	9.4	**4.0**	75.0
Left Ulnar			
Wrist—ADM	3.0	**4.1**	
Bel elb—wrist	8.0	**4.5**	**48.0**
Ab elb—bel elb	9.8	**4.6**	61.0
Right Tibial			
Ankle—ab Hal	4.4	5.3	
Pos. 2 ankle	15.2	4.2	41.0
Right Peroneal			
Ankle—EPB	5.6	5.6	
Fib head—ankle	15.2	4.1	40.0
Sensory Nerves:			
Right radial			
Forearm—thumb	2.7	**7.0**	55.0
Left radial			
Forearm—thumb	2.5	**6.0**	51.0
Left Median Anti			
Wrist—Dig III	4.6	**6.0**	52.0
Right Sural			
Calf—ankle	3.8	**5.0**	42.0
Right Hand Sens Anti			
Wrist—Dig III	4.2	6.0	50.0
Wrist—Dig V	3.7	7.0	52.0
Left Ulnar Anti			
Wrist—Dig V	**NR**		

Abbreviations: Ab elb—above elbow, ab Hal—abductor hallucis, ADM—abductor digiti minimi, Anti—antidromic, APB—abductor pollicis brevis, Bel elb—below elbow, Dig—digit, EPB—extensor pollicis brevis, NR—not recordable, Pos. 2 ankle—posterior to ankle.

Muscle and nerve biopsies (at age 6 years) reported mild muscle fibre size variation, type 1 fibre predominance with relative loss of type 2 fibres consistent with disuse. There was no lipid accumulation. The nerve biopsy revealed that some of the larger fibres had slight thinning of the myelin sheaths.

Genetic testing for the androgen receptor repeat expansion, *PMP22* duplication and next‐generation sequencing 76 gene inherited neuropathy panel had previously returned negative. Whole exome sequencing revealed two variants in *HADHA*: c.919‐2A>G, a pathogenic splicing variant and c.1003G>A, p.(Glu335Lys), a missense variant of uncertain significance. Parental testing confirmed that the patient was compound heterozygous for the two variants.

**FIGURE 1:**
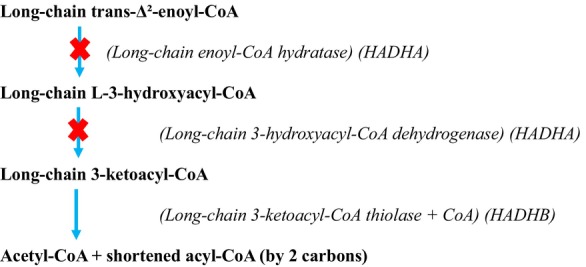
β‐Oxidation pathway for long‐chain fatty acids: Our patient is heterozygous for a pathogenic class 1 splicing variant, c.919‐2 A>G, and carries a second variant of uncertain significance, c.1003 G>A, p.Glu335Lys, in the ***HADHA*** gene. In the second step of fatty acid oxidation, the enzyme long‐chain 3‐hydroxyacyl‐CoA dehydrogenase oxidises the hydroxyl (OH) group to a keto group. Elevated levels of OH‐containing derivatives may indicate a defect at this step.

Plasma acylcarnitine profile was repeated; on this occasion, it identified a mild increase in medium and long chain acylcarnitines, including C16:1‐OH. Free carnitine levels remained within the normal range. The proteins encoded by *HADHA* and *HADHB* and their roles in the β‐oxidation pathway of long‐chain fatty acids are shown in Figure 1. Fatty acid oxidation flux was measured in cultured skin fibroblasts, demonstrating a skewed ratio of myristate to oleate, confirming mild mitochondrial trifunctional protein deficiency (Table 2).

**TABLE 2:** Fatty acid oxidation flux analysis in a skin biopsy (tritium release assay of β‐oxidation of fatty acids).

Date	Myristate (% of simultaneous controls)	Palmitate (% of simultaneous controls)	Oleate (% of simultaneous controls)
08/08/23@37°C	139	139	99
15/08/23@37°C	123	120	56
22/08/23@37°C	94	84	35
31/08/23@41°C	68	53	22

*Note:* Lower activity achieved with oleate than with the other two substrates. The ratio of myristate:oleate is skewed. This pattern consistent with (mild) mitochondrial trifunctional protein deficiency. The lower activities achieved at the higher temperature indicate that the variant could be temperature sensitive.

The p.(Glu335Lys) variant is reported twice in Clinvar as a variant of uncertain significance. It affects a highly conserved amino acid, is predicted damaging by in silico software PolyPhen (possibly damaging), SIFT (deleterious) and MutationTaster (disease causing) and is present at extremely low frequencies in available genome databases (absent from Exome Sequencing Project, 0.000022 in gnomAD and 0.000012 in 1000Genome project). As per ACMG criteria and taking into account the fatty acid oxidation flux results (PS3, PM2, PM3, PP3) this variant is now classified as likely pathogenic.

c.919‐2A>G is predicted to disrupt the highly conserved acceptor splice site of exon 10; it is predicted to be disease causing by MutationTaster. Clinvar lists this variant as pathogenic and likely pathogenic; it has been described as disease causing by multiple authors and is present at extremely low frequencies in available genome databases (0.000077 in Exome Sequencing Project, 0.00004 in gnomAD and absent from 1000Genome project). As per ACMG criteria, this is classified as pathogenic (PVS1, PM2, PP3, PP5).

At last review, although his long‐chain hydroxy species were within reference limits, the patient did not report any symptomatic improvement with dietary modification.

## 3 Interpretation

We describe a patient with MTPD with isolated sensorimotor neuropathy mimicking CMT as the first and only presenting symptom. While he later reported episodic worsening with activity and had one significant neuropathic deterioration following infection, there was no evidence of rhabdomyolysis, myopathy or metabolic decompensation, in contrast to most reported cases of patients with the neuromyopathic form of MTPD.

The neonatal form is the most prevalent and lethal, presenting with heart failure and early death. Hydrops fetalis, Reye‐like symptoms and pigmentary retinopathy may also be seen. Next most frequent is the hepatic form with onset from the neonatal period to 18 months, presenting with recurrent episodes of hypoketotic hypoglycaemia, hepatomegaly, encephalopathy with lethargy, seizures and liver failure; sudden death may occur. The neuromyopathic phenotype is rarest, usually presenting with progressive peripheral neuropathy with superimposed bouts of severe muscle weakness, myalgia and rhabdomyolysis triggered by prolonged exercise, illness, fasting and other metabolic stressors. Spiekerkoetter et al. reported that the combination of episodic rhabdomyolysis and peripheral neuropathy was present in 91% of patients with neuromyopathic MTPD [2]. Occasionally, episodic worsening occurs later in the course of the disease. There can be some overlap between the three phenotypes; skeletal muscle involvement may occur at any age.

The underlying pathophysiology of neuropathy in MTPD is not fully understood, but axonal degeneration with secondary myelin loss has been demonstrated on nerve biopsies [2]. The MTP complex plays a role in mitochondrial health, and the alpha subunit is involved in cardiolipin remodelling, important for maintaining mitochondrial membrane integrity [4]: axonal neuropathy is a common feature of mitochondrial disease. In addition, it is thought that accumulating acylcarnitines may be toxic to Schwann cells.

Only a small number of *HADHA*‐associated MTPD patients have been reported presenting with isolated neuropathy. Grunert et al. described four patients with isolated neuropathy due to MTPD, which was missed during NBS [3]. Three were siblings, two of whom were asymptomatic but had sensory neuropathy on NCS. The older sibling had an axonal sensory neuropathy, symptom onset at 4 years, but had elevated CK and transaminases on several occasions, with metabolic decompensations from 6 years. The fourth patient in this series had delayed developmental milestones with pes cavus and axonal sensorimotor neuropathy without metabolic decompensations by age 9 years. In another series of 11 patients with the neuromyopathic phenotype, only one had not developed rhabdomyolysis by last follow‐up (age 5 years); the remainder had first myoglobinuria by 13 years [2]. Four patients from three families with *HADHB*‐associated MTPD have been reported presenting with early onset axonal neuropathy. Hong et al. reported two Korean siblings presenting with childhood‐onset intermediate velocity neuropathy resembling CMT; both were walker‐dependent in early teens [5]. Lu et al. reported two unrelated patients of Chinese descent with infantile‐onset axonal neuropathy [6]. Several authors have demonstrated that patients with the milder neuromyopathic phenotype of MTPD may have absent or only mild biochemical abnormalities. Specific enzyme activity does not correlate well with phenotype; however, fibroblast fatty oxidation flux assays show correlation with phenotype when expressed as a ratio of percentage activity of myristate/oleate, with higher values seen in severe neonatal onset disease and lower values seen in the neuromyopathic form. Our patient's myristate/oleate ratio at 37° was 1.4, slightly lower than previously reported neuromyopathic patients [7] providing a hypothesis for his milder phenotype without rhabdomyolysis, presumably due to residual enzyme activity and possibly other modifying cellular factors in vivo.

Fatigability as seen in our patient was not described in any of the previously reported patients with an isolated neuropathic phenotype. Moreover, the improvement with alcohol is a unique feature not previously reported with MTPD. Alcohol and in particular beer, our patient's preferred alcoholic drink, is high in simple carbohydrates which are readily available for metabolism due to the simple sugars contained. Carbohydrates are an important source of energy in patients with MTPD as the goal is to minimise reliance on fatty acids for energy production. Neuropathic decompensations without rhabdomyolysis or other metabolic decompensation such as occurred in our patient have been reported rarely. In such cases, patients have shown slow improvement afterwards, similar to this case [3].

Neuropathy in MTPD is usually length‐dependent. Neurophysiology is usually axonal and sensorimotor, but pure motor or sensory only patterns have also been observed [3]. In our patient, neuropathy was axonal and sensorimotor, but non‐length dependent.

CK is typically significantly raised (up to 165 000 IU/L) during the episodes of acute decompensation [2]. The first attack of rhabdomyolysis may occur between 1 and 13 years of age [2]. Our patient has a persistently mildly elevated CK, which is frequently seen in chronic neuropathy, and which has not changed significantly over the years or during exacerbations. During metabolic decompensation, blood tests may show hepatic dysfunction, hypoketotic hypoglycaemia (in 78% cases), high ammonia and lactic acidosis. None of these were seen in our patient during his single episode of decompensation requiring hospitalisation. Metabolic blood tests may show elevated plasma levels of non‐oxidised medium‐chain and long‐chain fatty acids. Acylcarnitine profile in dried blood spots (DBS) by tandem mass spectrometry (MS/MS) reveals increased levels of long‐chain 3‐hydroxyacylcarnitines (C14OH, C16OH, C16:1OH, C18OH, C18:1OH) and decreased free carnitine (C0). Organic acids dicarboxylic and 3‐hydroxy‐dicarboxylic acid may be present in urine. These metabolic abnormalities are not always seen, however [2], and our patient did not show any of the above abnormalities when tested.

Definitive diagnosis of MTPD can be made by enzymatic analysis in cultured skin fibroblasts and/or molecular genetic analysis. LCHAD and LCKT activities are typically below 40% of normal, although enzyme levels do not correlate with the severity of symptoms [1]. Substantial molecular heterogeneity is seen in both α and β subunit variants causing MTPD. However, phenotype–genotype studies have suggested that frameshift, nonsense or splicing variants typically cause severe phenotypes, whereas missense variants generally cause milder phenotypes. Our patient was compound heterozygous for a known pathogenic splicing variant along with a missense variant of uncertain significance p.(Glu335Lys). Enzymatic analysis subsequently was consistent with mild mitochondrial trifunctional protein deficiency, confirming that this missense variant is pathogenic.

In conclusion, we have reported a patient with mild MTPD presenting with a very rare isolated neuropathic phenotype, initially mimicking CMT, and have confirmed the pathogenicity of the p.(Glu335Lys) variant. *HADHA* and *HADHB* are not included on most neuropathy gene panels, so the diagnosis can easily be missed if whole exome/genome sequencing is not performed in a patient with apparent inherited neuropathy. Given that dietary management may prevent some complications of MTPD, achieving a diagnosis early is important. Metabolic testing should be considered in patients presenting with isolated neuropathy where an alternate cause has not been identified.

## Conflicts of Interest

The authors declare no conflicts of interest.

## Data Availability

The data that support the findings of this study are available on request from the corresponding author. The data are not publicly available due to privacy or ethical restrictions.
